# Recent advances in immunoassays and biosensors for mycotoxins detection in feedstuffs and foods

**DOI:** 10.1186/s40104-021-00629-4

**Published:** 2021-10-11

**Authors:** Runxian Li, Yang Wen, Fenglai Wang, Pingli He

**Affiliations:** State Key Laboratory of Animal Nutrition, College of Animal Science and Technology, China Agricultural University, Beijing, 100193 China

**Keywords:** Biosensors, Immunoassays, Multiple detection, Mycotoxins, Nanomaterials, Rapid detection

## Abstract

Mycotoxins are secondary metabolites produced by fungus. Many mycotoxin species are highly toxic and are frequently found in cereals and feedstuffs. So, powerful detection methods are vital and effective ways to prevent feed contamination. Traditional detection methods can no longer meet the needs of massive, real-time, simple, and fast mycotoxin monitoring. Rapid detection methods based on advanced material and sensor technology are the future trend. In this review, we highlight recent progress of mycotoxin rapid detection strategies in feedstuffs and foods, especially for simultaneous multiplex mycotoxin determination. Immunoassays, biosensors, and the prominent roles of nanomaterials are introduced. The principles of different types of recognition and signal transduction are explained, and the merits and pitfalls of these methods are compared. Furthermore, limitations and challenges of existing rapid sensing strategies and perspectives of future research are discussed.

## Introduction

Topics related to food safety have been attracting significant attention all over the world. Contaminants in cereals and animal products are related closely to human health. Mycotoxins are secondary metabolites of fungus and may be produced during growth, production, processing, and storage of cereals, grains and feedstuffs [1]. There are over 300 mycotoxins identified, many of which are extremely toxic and difficult to degrade by cooking, baking or frying because of their high heat-stability [2]. The International Agency for Research on Cancer (IARC) has classified several mycotoxins into different categories based on their carcinogenic risk to human health. For example, aflatoxin B1 (AFB1) is the most carcinogenic mycotoxin and is classified as Group 1 while ochratoxin A (OTA) and fumonisin are classified into Group 2B as possibly carcinogenic in humans [3–5]. Many countries have developed standards to control mycotoxin contamination of food for safety reasons [6]. In China, maximum residue levels (MRLs) for mycotoxins in various foods have been strictly regulated in national food safety standard system. Consequently, a powerful detection method for mycotoxins is critical component in assuring food safety.

Traditional analytical methods such as enzyme-linked immunoassay (ELISA), thin layer chromatography, gas chromatography or high-performance liquid chromatography (HPLC) coupled with ultraviolet detection, fluorescence detector electron capture detectors, diode array detectors, and mass spectrometry (MS) detectors have been used to detect mycotoxins for decades [7–10]. Most of these traditional methods are accurate and sensitive but time-consuming, expensive, or require sophisticated instruments and professional technicians. They are not appropriate for a large number of samples and on-site screening. Because of demands from food industry professionals and government regulations, analytical methods that can detect multiple mycotoxins simultaneously and are fast, simple, sensitive, and low-cost with high-throughput have become the norm in the field of food safety and will continue into the future [11].

Rapid detection technology is a concept relative to traditional analysis methods and laboratory detection technology. It is often based on interdisciplinary subjects such as nanomaterials science, immunology, molecular biology, spectroscopy, and electrochemistry. Rapid detection is simple, cheap, easy to operate, and only requires portable instrument and a very short detection duration. It enables detection method to meet the need of the real-time on-site mycotoxins screening in the field of food safety. Herein, we review published research reports on rapid detection technology for mycotoxins from 2016 to 2021 including immunoassays and biosensors. Principles of recognition and signal transduction strategies are explained, and the pros and cons compared to existing method are discussed. Especially, we highlight studies on simultaneous detection of multiple mycotoxins. Limitations, challenges and perspectives of the future developments in this field are discussed.

## Types of recognition strategies

Specific recognition of analytes is the primary step of rapid detection, which ensures the specificity and selectivity of the analytical method. In this section, different types of recognition elements for mycotoxin detection are introduced including antibodies, aptamers, and molecularly imprinted polymers (MIPs).

### Antibodies

Antibodies are immunoglobulins produced by the immune system that specifically bind to the corresponding antigens. Based on the preparation processes used, antibodies are classified into polyclonal, monoclonal, or recombinant categories. Antibody-antigen recognition is regarded as a gold standard because of its properties of high specificity and affinity. Antibodies and antigens are the most widely commercialized recognition and capture agents applied in immunoassays and biosensors for clinical diagnosis, disease treatment, environment monitor, and food safety control [12–15]. However, there are obvious disadvantages of using antibodies. First, acquisition of high-quality antibodies requires immunization and purification which is a complex process that is time-consuming and costly. Second, antibodies are sensitive to pH and temperature conditions, which may narrow the practical application. Finally, only immunogenic and immunoreactive molecules can be identified by antibodies. Targets of small molecular weight need to conjugate with a carrier protein to enhance immunogenicity, but the chemical conjugation efficiency of mycotoxins to a protein carrier is low.

To overcome these limitations of antibodies, variable region of heavy chains antibody (nanobody) and phage-displayed mimotope peptides may be some utility in solving these antibody problems [16]. Nanobody as a naturally deficient light chain antibody is highly stable and small in size, which makes it easy to conserve and use. Phage displayed mimotope peptide can simulate the epitope of the target analytes so the hapten-carrier conjugate can be replaced by a mycotoxin-free mimic which also makes fabrication of green sensors possible [17–21].

### Aptamers

Aptamers are small fragments of oligonucleotide sequences (single stranded DNA or RNA), which usually contain 10 to 100 bases. Aptamers bind to their targets by folding into specific three-dimensional structures. They are selected from a combinatorial DNA library by a technology named systematic evolution of ligands exponential enrichment (SELEX) and can bind to various targets ranging from ions to cells. Aptamers are usually considered to be ideal affinity reagents alternatives to antibodies. Compared to antibodies, aptamers are inexpensive, stable, reversible, not limited by immunogenicity of targets, and do not require immunization of animals during production. Aptamers have high bioavailability and are easy to modify. As an excellent alternative to antibodies, aptamers are being deployed in biosensor, disease diagnosis, and drug delivery [22–24]. However, acquiring aptamers with high affinity and sensitivity especially for small molecules is relatively inefficient. Over the past few years, selection techniques have been improved constantly. A series of novel SELEX-based techniques such as Cell-SELEX, Capillary electrophoresis-SELEX, Capture-SELEX, and Post-SELEX modifications have been developed. Compared with the original SELEX technique, aptamers developed with these new approaches display high affinity and specificity while selection processes are more efficient and cost-effective [25, 26]. Although aptamers are currently prevalent in scientific literatures, commercial aptamer-based mycotoxin detection kits are not yet available [27].

### Molecularly imprinted polymers (MIPs)

Molecular imprinting is a technology developed based on bionic science and simulation of the interactions of enzymes with their target substrates and receptors with their antibodies in nature. Molecularly imprinted polymers (MIPs) are obtained by mixing imprinted molecules (template molecules) with appropriate functional monomers (usually small molecular compounds) and crosslinking agents. Then polymerize them and eluting the imprinted molecules by appropriate methods. Molecularly imprinted polymers have been called “artificial antibodies”. They are preserved easily, resistant to extreme conditions such as high temperature, high pressure, acid, and alkali, and difficult to destroy by biodegradation [28]. Consequently, MIPs are widely applied in optical sensors, electrochemical sensors, quartz crystal microbalances, simulated catalysis, membrane separations and solid phase extractions (SPE) [29–33]. In spite of these benefits, MIPs face several challenges in practical applications. A considerable drawback of MIPs is unspecific interactions, especially with small molecules, which requires non-imprinted polymers (NIPs) be employed to characterize nonspecific binding [34]. Selectivity and binding affinity of MIPs still require development to allow detection of target compounds in complex matrixes [33]. Commercialization of MIPs is in its infancy. Relatively few research studies focused on MIPs-based mycotoxin sensors are reported in the scientific literature.

## Rapid detection strategies

### Immunoassays

Immunoassays are based on immunological principles that use antibodies to recognize and capture target antigens or haptens. Enzyme-linked immunosorbent assays (ELISA) are early immunoassays that have been commercially applied in mycotoxin detection with good sensitivity and accuracy [35]. Enzyme-linked immunosorbent assays are simpler than instrumental methods such as HPLC and LC-MS/MS, but they still require complex operations and several hours of incubation and microplate washing procedures. Therefore, researchers have been working to develop various immunoassays for better analytical performance. New procedures such as fluorescent-linked immunosorbent assays (FLISA) and chemiluminescence enzyme immunoassays (CLEIA) can reduce detection duration and improves the sensitivity by relying on enhanced optical signals [36–38]. Lateral flow immunoassays (LFIA) and immunosensors have become popular analytical methods in recent years. In this part, LFIA is chiefly introduced and immunosensor is introduced in the following sensor section.

#### Lateral flow immunoassays

Lateral flow immunoassays (LFIA), also known as immunochromatographic assays (ICA), are the most frequently used and commercialized rapid sensing platform and widely applied in disease diagnosis, point-of-care testing (POCT) and food safety monitoring because of its low cost, rapidity and simplicity [39, 40]. The detection process is integrated on a small strip and the signal analysis requires portable instruments and very little time. Lateral flow immunoassays are highly convenient to achieve simultaneous detection of multiple mycotoxins by using multiple test lines and signal labels. There are two main detection principles used, sandwich assay (two types of antibodies are required) and competitive assay (one type of antibodies are required). Mycotoxin cannot acquire two types of recognition antibodies because it doesn’t own multiple epitopes, so the competitive LFIA is preferred.

High-quality antibodies and antigens are primary guarantees for the high sensitivity and specificity of LFIA. However, the recognition of limited immunogenic targets by antibodies has hindered development of LFIA in several fields of study. Secondly, there is a contradiction in the competitive detection mode itself. That is, within a certain range, the less quantity of antibodies makes the competition between free target analytes and the immobilized antigens more effective on the test zone. So, the lower the specific antibody concentration, the lower the detection limit. However, the amount of the label conjugated with antibody is reduced simultaneously with the low concentration of antibody, which decreases signal intensity of the test line and hinders successful detection of the targets.

To overcome these shortcomings above, researchers developed better-performed antibodies or signal labels with high luminescent intensity and stability [41]. Besides, the new detection principle based on fluorescence quenching that can directly response to changes of target analytes has been attempted [42].

#### Signal labels

Gold nanoparticles (AuNPs) are the most frequently used signal labels in LFIA because they are easy to synthesize, inexpensive, and emit visible red to purple light when they aggregate. However, an obvious drawback of AuNPs-based LFIA is the instability and weak optical intensity of AuNPs resulting in low sensitivity [41, 43]. Recently, non-spherical, multilayer AuNPs with varying colors such as flower-like AuNPs have been used in LFIA instead of traditional spherical AuNPs to improve the sensitivity [44–46]. Wu et al. [47] engineered gold nanoparticles to create multicolor labels such as red gold nanospheres, purple gold nanocacti, blue gold nanoflowers and black hyperbranched Au plasmonic blackbodies. Four mycotoxins (FB1, ZEN, OTA and AFB1) can be successfully detected in corn using this multicolor LFIA strip and limit of detection (LOD) for FB1, ZEN, OTA, and AFB1 were 3.27, 0.70, 0.10, and 0.06 ng/mL, respectively.

Use of various fluorescent labels with higher stability and signal intensity in LFIA to enhance the sensitivity has become commonplace. Quantum dots (QDs) are semiconductor nanoparticles that possess excellent optical properties including size-tunable emission, broad adsorption, narrow photoluminescence spectra, high photostability, large Stokes’s shift, and long fluorescence lifetime [48]. Fluorescent microspheres (FMs) are polymers that contain numerous fluorescent particles such as fluorescent dyes and quantum dots in the nanobeads. Fluorescent microspheres are more stable and have substantially higher fluorescent intensity than that of single fluorescent molecule [49]. Carbon-based nanoparticles (CNPs) such as carbon dots have desirable optical properties similar to quantum dots, and have low toxicity [50]. These materials are promising LFIA labels because they are water soluble and they are not easy to aggregate and precipitate. Functional groups like carboxyl can be easily modified on their surfaces, which helps them bind to antibodies with great stability due to covalent binding between hydroxyl and carboxyl groups.

**Fig. 1:**
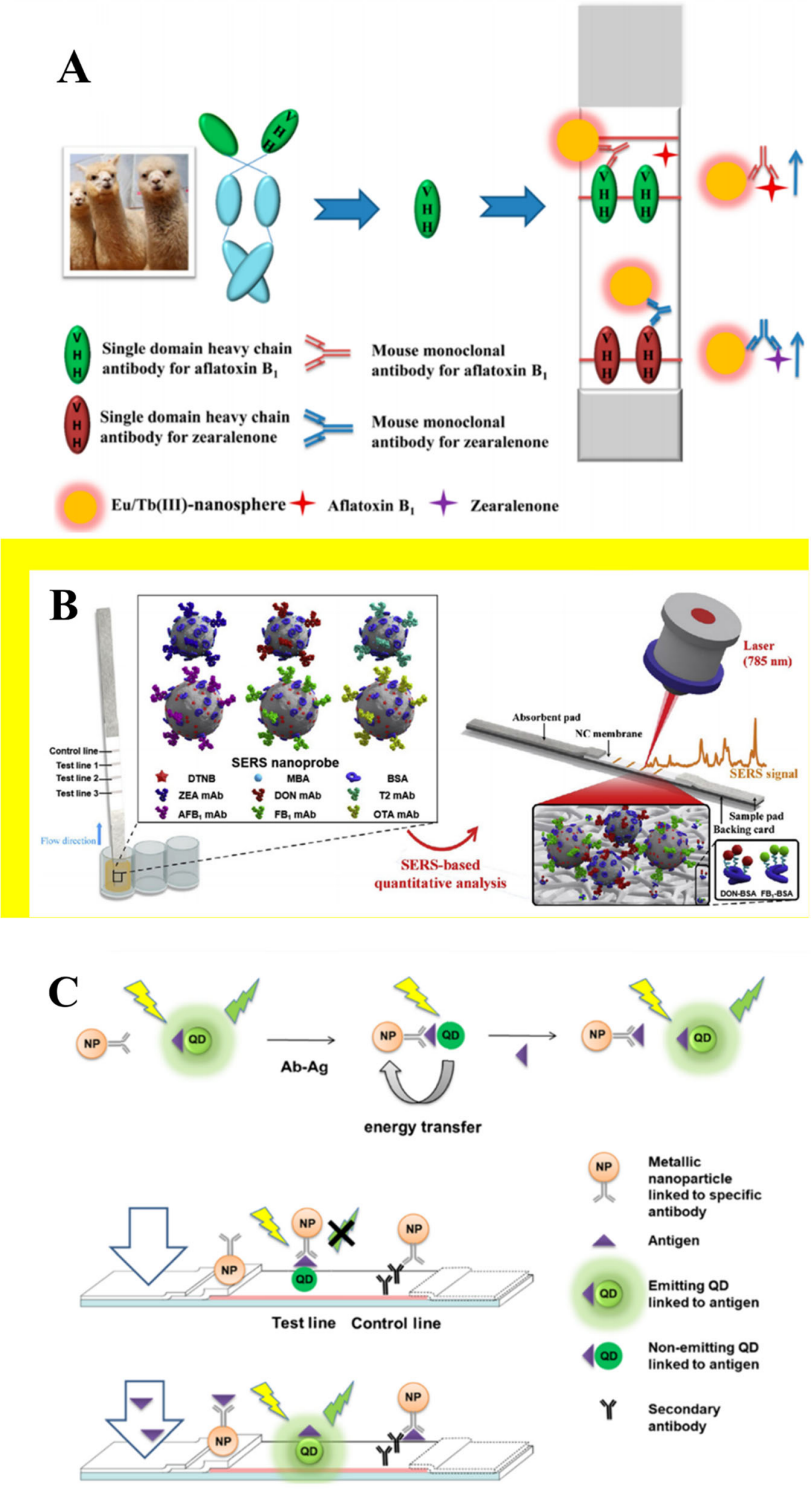
Structure and test procedure of LFIA. (A) Time-resolved fluorescent Eu/Tb (III) nanosphere for detecting AFB1 and ZEN; (B) multiplex SERS-based lateral flow immunosensor for the detection of six mycotoxins; (C) detection of OVA based on donor probe (CD-OVA) and accepter probe (AgNP-Ab). Diagrams A, B, and C are adapted from Ref. [52] Ref. [60] and Ref. [42], respectively

Time-resolved fluorescent microspheres (TRFMs) are recently employed as signal labels frequently. Lanthanides such as Eu (III), Tb (III) have much longer fluorescence lifetime than the previously mentioned fluorescent materials so that they can eliminate the background fluorescence interference from the matrix, thus improving the sensitivity of LFIA [51–53]. For example, Fig. 1A shows time-resolved fluorescent Eu/Tb (III) nanosphere and idiotypic nanobodies for multiplex detection of two mycotoxins simultaneously in maize [52]. Sensitivity of LFIA was improved by the enhanced fluorescence of Eu/Tb (III) nanosphere with the LOD of 0.05 and 0.07 ng/mL for AFB1 and ZEN, respectively. Another study reported novel α-Fe_2_O_3_ nanocubes used as LFIA label for simultaneously detecting two mycotoxins. These nanocubes have been employed to detect AFB1 and DON residues in mung bean, millet and corn, with the visual LOD for AFB1 and DON of 0.01 and 0.18 ng/mL, respectively [54].

Researchers have compared various signal labels to provide a reference for selection of appropriate labels. For instance, colloidal gold (CG) and QDs as labels for multi-mycotoxin detection in cereal matrices have been compared. The QD-LFIA was more sensitive and economical than CG-LFIA [41]. In another study, CG and FMs were compared as the LFIA labels for T-2 toxin detection in rice, fresh milk, and chicken feed. The cut-off values of the CG-LFIA and the FMs-LFIA were 400 μg/kg and 100 μg/kg, respectively. And they found CG and FMs performed differently in different matrixes. Under the same experimental conditions, CG had better tolerance to milk while FMs had better tolerance to chicken feed [55]. A comparative study of LFIA of ZAE in cereals using CG, QDs and polystyrene microspheres (PMs) as signal label was conducted [56]. Researchers reported that QD-LFIA and PM-LFIA were ten times more sensitive than CG-LFIA. Besides, a smartphone-based dual mode LFIA device for multiplex mycotoxins in cereals was developed, which was integrated with AuNPs and TRFMs as labels. The result indicated that the LOD of TRFMs mode were lower than that of AuNPs mode [57].

Gold nanoparticles and sliver nanoparticles can provide another detectable signal termed Surface-Enhanced Raman Spectroscopy (SERS). Surface-Enhanced Raman Spectroscopy effect is the phenomenon that in the excitation region of some specially prepared metal good conductor surface or colloidal sol. The Raman scattering signal of adsorbed molecules is greatly enhanced compared with the ordinary Raman scattering signal because the enhanced electromagnetic field on the sample surface. When SERS nanotags (gold, silver or Au-Ag alloy nanoparticles labelled with Raman reporter molecules) are exposed to a laser light source, the incident field is obviously enhanced at active sites (electromagnetic “hot spots”) due to localized surface plasmon resonance effects [58, 59]. Combining SERS detection and lateral flow immunoassay is also regarded as a promising choice for highly sensitive and multiplex mycotoxin detection. Fig. 1B show a SERS-based LFIA combined dual-Raman label (DTNB and MBA) with triple-line LFIA and labelled-Au@Ag core-shell nanoparticles serving as SERS nanoprobes [60]. The SERS-based LFIA can be used for the detection of six common mycotoxins (AFB1, ZEN, FB1, DON, OTA and T-2) simultaneously in maize with high sensitivity and selectivity within 20 min. Signal acquisition requires only a portable Raman System.

#### Fluorescence quenching LFIA

In addition to competitive assays, researchers have explored LFIA based on fluorescence quenching principle to improve sensitivity. Competitive assays need many target analytes to compete with Immobilized antigen then make the signal of test lines “turn off”. The signal is an indirect signal, which leads to relative low sensitivity. While the fluorescence quenching LFIA can provide a “turn on” signal, which responses to the amount of target analytes directly [61, 62]. Inner-filter effect (IFE) and Förster resonance energy transfer (FRET) are widely used quenching mechanisms. Quenching agents usually are metal nanoparticles such as AuNPs or AgNPs. Inner-filter effect is a radiation energy transfer mechanism while Förster resonance energy transfer is a non-radiative process. Quenching effects occur when the absorption spectrum of the accepter (quencher) overlaps with the donor’s excitation or emission spectra. Antigens (analytes) are usually linked with fluorescence donors then immobilized on the test line. Antibodies are usually linked with fluorescence accepters (quenchers) which can bind to the analytes. If there is no analyte in the sample solution, antibodies-linked accepters will bind to antigens-linked donors resulting in fluorescence quenching of the donor. Conversely, if there are many analytes in the sample solution binding with antibodies-linked accepters, the donor’s fluorescence will be restored. Fig. 1C shows a fluorescence quenching LFIA based on the CdSe/ZnS quantum dots and gold/silver nanoparticles for straightforward detection of fumonisin in maize flour [42]. Silver nanoparticles overlapped excitation bands of the QDs, and AuNPs overlapped emission bands. The QD quenching was due to IFE. Silver nanoparticles were demonstrated to be more efficient in QDs quenching because absorbing the exciting light was more efficient in IFE. Compared to conventional AgNPs-based LFIAs, the visual detection limit was four times lower. Another attempt explored a quencher system based on FRET mechanism for fluorometric LFIA for zearalenone detection in cereal samples. Carbon dots were conjugated to ovalbumin as the donor signal probe and AgNPs-Antibody served as the acceptor signal probe. The LOD of the LFIA was 10 times better than the “turn-off” AgNPs-based LFIA [63].

Based on existing studies, fluorescence quenching LFIAs exhibited a better performance than traditional AgNPs-based LFIAs but there have been hardly any researches on multiple mycotoxin detection using fluorescence quenching LFIA. Most of traditional LFIAs and fluorescence quenching LFIA can only render qualitative or semi-quantitative results at present. False positive and false negative results are still a problem. Selected studies of LFIAs for mycotoxin detection published in recent years are listed and compared in Table 1.

**Table 1:** Summary of LFIA strips for detecting mycotoxins

Target	Principle	Signal material	Sample	LOD	Ref.
FB1/ZEN/OTA/AFB1	Competitive LFIA	Au nanospheres, Au nanocacti, Au nanoflowers and hyperbranched Au plasmonic blackbodies	Corn	3.27/0.70/0.10/0.06 ng/mL	[47]
FB1/DON	Competitive LFIA	Flower-like gold nanoparticles	Chinese traditional medicine	5.0/5.0 ng/mL	[45]
FB1/DON	Competitive LFIA	Au nanospheres/Au nanoflowers	Grain	20/5 ng/mL	[44]
DON/AFB1	Competitive fluorescent LFIA	α-Fe_2_O_3_ nanocubes	Food	0.18/0.01 ng/mL	[54]
CIT/ZEN	Competitive fluorescent LFIA	Europium nanoparticles	Corn	0.06/0.11 ng/mL	[64]
ZEN/DON	Competitive fluorescent LFIA	Near-infrared dyes	Maize	0.55/3.8 μg/kg	[65]
ZEN/OTA/FB1	Competitive fluorescent LFIA	Quantum dot nanobeads	Wheat	5/20/10 ng/mL	[66]
ZEN/DON	Competitive fluorescent LFIA	Silanized quantum fots	Maize and wheat	40 and 400 μg/kg (Cutoff value)	[67]
AFB1/ZEN/DON/T-2/FB1	Competitive fluorescent LFIA	Time-resolved fluorescence microspheres	Cereals	0.42/0.10/0.05/0.75/0.04 μg/kg	[57]
AFB1/ZEN	Competitive fluorescent LFIA	Time-resolved fluorescence microspheres	Maize	0.05/0.07 ng/mL	[52]
AFB1/ZEN/DON	Competitive fluorescent LFIA	Quantum dot microbeads	Feedstuff	10/80/500 pg/mL	[68]
DON/T-2/ZEN	Competitive fluorescent LFIA	Amorphous carbon nanoparticles	Maize	20/13/1 μg/kg	[69]
AFB1/FB1/OTA	Competitive fluorescent LFIA	Quantum dot nanobeads	Cereals	1.65 pg/mL, 1.58/0.0059 ng/mL	[70]
DON	Competitive fluorescent LFIA	Polydopamine coated zirconium metal-organic frameworks	Meat	0.18 ng/mL	[71]
AFB1	Fluorescence quenching LFIA (IFE)	Flower-like gold nanoparticles/quantum dots	Soybean sauce	0.004 μg/L	[61]
FB1	Fluorescence quenching LFIA (IFE)	Sliver nanoparticles/quantum dots	Maize flour	62.5 μg/kg	[42]
ZEN	Fluorescence quenching LFIA (FRET)	Sliver nanoparticles/carbon dots	Cereals	0.1 μg/L	[63]
AFB1/ZEN/FB1/DON/ OTA/T-2	SERS-based LFIA	DTNB and MBA labelled Au@Ag coreshell nanoparticles	Maize	0.96/6.2/0.26/0.11/15.7/8.6 pg/mL	[60]

### Biosensors

Biosensors are detection devices that can convert the information measured into electrical signals or other information output of the required form. The biosensor system mainly consists of a biorecognition (biosensing) element and a transducer element. Biorecognition elements such as antibodies, antigens, nucleic acids, and enzymes are used to identify and sense target analytes. Transducer elements convert the physical quantity signal of generated by the recognition elements into detectable signals. According to the principle of the signal transduction, biosensors can be classified into electrochemical, optical, mass-sensitive, and thermal sensors. They have become vital and powerful analytical tools widely applied in various fields including medicine, food, agriculture, environment, and industry. Biosensor systems were introduced to enhance food safety in the 1980s and provide great commercial potential currently [16, 72–74]. In this section, electrochemical and optical biosensors for mycotoxin detection are mainly introduced.

#### Optical biosensors

Optical sensors rely on variation in optical signals generated by transducer from molecular recognition events on sensing element. This approach excels in superiorities of simplicity, speed of detection, sensitivity and visualization [75]. On the basis of optical signals, optical biosensors are divided into many subclasses including colorimetric, fluorescent, chemiluminescent and surface plasmon resonance. Optical biosensor systems also exhibit many possibilities for determination of multiple mycotoxins simultaneously.

#### Colorimetric biosensors

Colorimetric biosensors are based on color changes induced by target analytes that can be easily distinguished by the naked eye. Gold or silver nanoparticles are most frequently applied as indicators to utilize their surface plasmon resonance (SPR) properties. The general principle is that recognized targets induce aggregation of AuNPs or AgNPs from well-dispersed state resulting in color change from red to blue.

A colorimetric aptasensor for simultaneously detecting ochratoxin A and aflatoxin B1 in peanut was reported [76]. Researchers fabricated a Fe_3_O_4_/GO based platform and a Fe_3_O_4_@Au based platform for AFB1 and OTA sensing. Quantitative detection of OTA and AFB1 was respectively carried out by release of thymolphthalein and 3,3′,5,5′-tetramethylbenzidine catalyzed by gold nanoparticles, respectively. In this work, OTA and AFB1 were easily and visibly detected in one system with the linear ranges of 0.5–80 and 5–250 ng/mL and did not interfere with each other because of different color reaction conditions.

In another attempt, a colorimetric sensor based on array of gold and silver nanoparticles was fabricated for simultaneous detection of AFB1, AFG1, AFM1, OTA and ZEN [77]. Mycotoxin interactions with nanoparticles induced aggregation of gold or silver nanoparticles and the color changed. Every type of mycotoxin was recognized by its unique colorimetric signatures with the LOD of 2.7, 7.3, 2.1, 3.3 and 7.0 ng/mL for AFB1, AFG1, AFM1, OTA and ZEN, respectively. The developed colorimetric method had the advantages of high throughput, simplicity, rapidity and low cost. This system was tested in pistachio, wheat, coffee, and milk samples. Most of colorimetric sensors suffer the weakness of low sensitivity which suggests optical signal intensities still need further enhancement.

#### Fluorescent biosensors

At present, fluorescent biosensors are the most popular optical sensors because of their high sensitivity, stable signal, and fast response. Various nano-materials such as magnetic nanoparticles (MNPs), quantum dots (QDs), carbon nanoparticles (CNPs), gold nanoparticles (AuNPs), fluorescent nanobeads, and graphene oxide (GO) have been widely applied in construction of fluorescent sensors [78–80]. Lanthanide-based luminescent materials such as upconverting nanoparticles (UCNPs) and time-resolved fluorescence materials (TRFMs) have also become popular in recent years [81, 82]. After target analytes have been identified by recognition elements, quantitative sensing of one mycotoxin is achieved by the variation of fluorescent signals based on fluorescence quenching, fluorescence enhancement, or displacement of fluorescent labels.

**Fig. 2:**
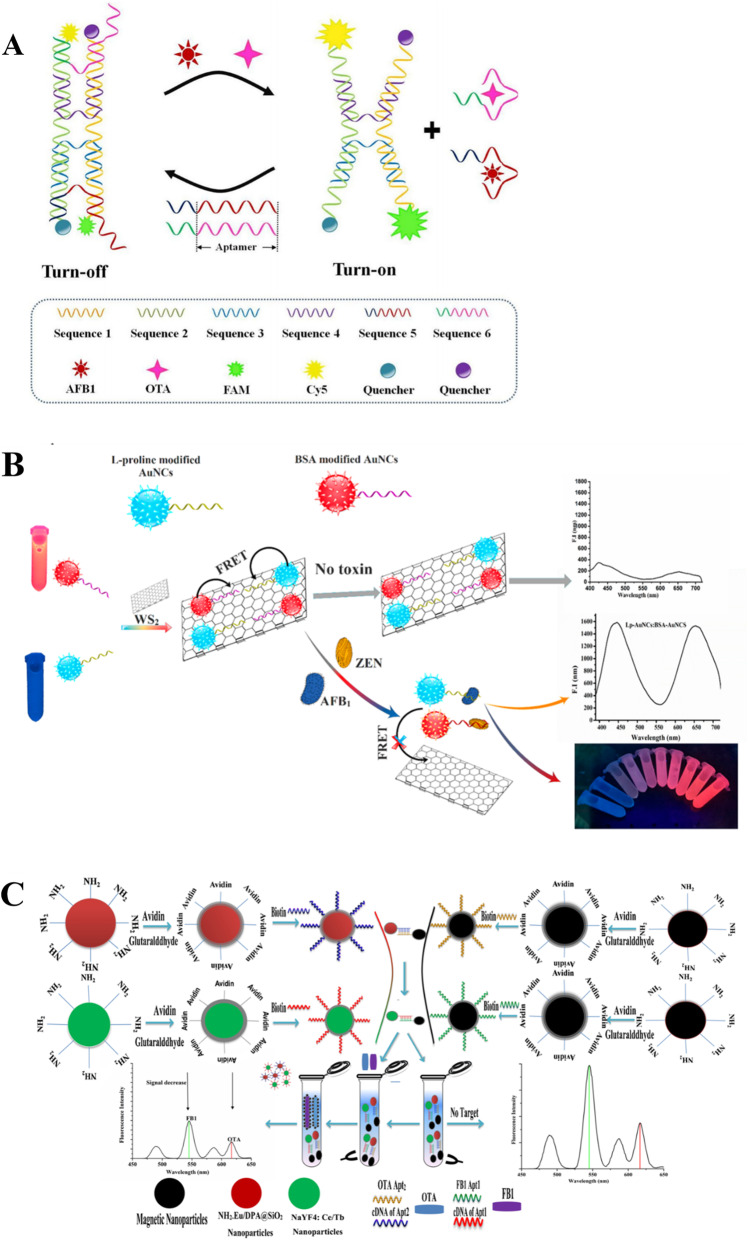
Schematic illustration of the optical sensing platforms. (A) Dual DNA tweezers nanomachine for simultaneous detection of AFB1 and OTA; (B) a FRET-based platform using WS_2_ nanosheet and dual-color gold nanoclusters (AuNCs) for simultaneous detection of AFB1 and ZEN; (C) a fluorescence aptasensor for FB1 and OTA detection by using TRF-NPs and MNPs; Diagrams A, B, and C are adapted from Ref. [83], Ref. [84], and Ref. [81], respectively

Figure 2A shows dual DNA tweezers nanomachine has been utilized for simultaneous detection of AFB1 and OTA [83]. The fluorophores were quenched by the quencher when DNA tweezers were closed. In the presence of AFB1 and OTA, DNA tweezers opened after the aptamer strands bind with their corresponding targets resulting in the activated fluorescent signals. The aptasensor exhibited satisfactory applicability in food samples including corn, peanut, olive oil, peanut oil, and coffee.

A FRET-based platform was developed by WS_2_ nanosheet and dual-color gold nanoclusters (AuNCs) for simultaneous detection of AFB1 and ZEN in maize [84]. Gold nanoclusters and WS_2_ were connected by AFB1/ZEN-aptamer-BSA resulting in fluorescent quenching of AuNCs. Fluorescence turned on when target analytes were present (Fig. 2B). The sensor achieved multiplex target determinations in a homogeneous system with excellent simplicity, sensitivity, and selectivity. However, this system can only provide semiquantitative detection. Another FRET-based platform was proposed using GO/Fe_3_O_4_ as a single energy acceptor, which can quench the dual aptamer-modified QDs simultaneously and eliminate background interference effectively by magnetic separation [85]. This system successfully detected AFB1 and FB1 in peanut with the LOD of 6.7 and 16.2 pg/mL, respectively.

Time-resolved fluorescence materials are also excellent materials for fluorescent probes design because of its long fluorescence lifetime, which provides anti-interference capability. For example, Fig. 2C shows a fluorescence aptasensor for FB1 and OTA detection in maize samples by using time-resolved fluorescence nanoparticles (TRF-NPs) and MNPs [81]. The aptamer-MNPs and cDNA-TRF-NPs formed the duplex structure. With the addition of FB1 and OTA, the aptamer-MNPs bound to FB1 and OTA then dissociated from its cDNA, leading to a decrease in fluorescent intensity when magnetic separation. The sensor was user-friendly with LOD for FB1 and OTA were 0.019 and 0.015 pg/mL, respectively. The aptasensor exhibited better practicability than common fluorescent system in complex metrics because of the unique merits of TRFM-NPs.

#### Chemiluminescence and electrochemiluminescence biosensors

Chemiluminescence is a light radiation phenomenon resulting from a chemical reaction, which is also an important tool for molecular sensing. But, low luminescence intensity of chemiluminescence needs to be enhanced by enzymes or metal nanoparticles in practical applications [86]. For instance, a AgNPs modified surface-plasmon-coupled chemiluminescence immunosensor was developed for aflatoxin B1 and ochratoxins A detection in red yeast rice [87]. The generated chemiluminescence signal on the chip was amplified by AgNPs through the SPR phenomenon, which greatly enhanced sensitivity of the biosensor. The chip array offered a high-throughput detection mode for multiple targets. But several incubations and washing operations were needed, which complicated the assay. The problem of uneven brightness made advanced instruments and equipment necessary to achieve acceptable readings.

Electrochemiluminescence (ECL), as a subclass of chemiluminescence or electrochemistry, is regarded as the reverse of photoelectron chemistry. Voltage or current serves as the excitation source and the generated chemiluminescence is used as the detectable signal. With ECL, the electro-active species generated in the vicinity of the electrode surface are in excited states emitting light that arises from the high-energy electron-transfer reactions of these species. Luminol and Ru (bpy)_3_^2+^ are classic ECL luminophores while QDs are beginning to be applied to ECL sensors to achieve higher efficiency and stability of ECL [88, 89]. Electrochemiluminescence-based sensors have fast response, high sensitivity and low background interference, which is a promising strategy for applying in rapid detection [90]. However, ECL biosensors have similar drawbacks observed with electrochemical biosensors, such as instability of electrode including the degradation biosensing reagents and the instable optical signal of ECL luminophores. And few ECL instruments have been reported. Advanced and portable equipment is urgently needed.

A nicking endonuclease-powered DNA walking machine was proposed to fabricate an ECL aptasensor for OTA detection [91]. Quantum dots produced ECL signal and was greatly amplified by DNA walking machine, which increased the sensitivity of this ECL sensor. This ECL aptasensor showed satisfactory recoveries of OTA in beer and wine with the LOD of 12 pmol/L. Other researchers developed a label-free ZnCdS@ZnS QDs-based ECL immunosensor for AFB1 detection in lotus seed [92]. Quantum dots and Nafion were assembled on Au electrode surface as ECL signal probes and anti-AFB1 antibodies were coupled as the capturing element. Using of Nafion and core-shell QDs greatly enhanced the ECL signal thus improved the sensitivity. The label-free design simplified the synthesis steps and reduced detection time.

#### Surface plasmon resonance biosensors

Surface plasmon resonance (SPR) sensors are optical sensors based on changes in the refractive index due to the mass changes that occur when molecules bind to the sensor surface. These changes in refractive index can provide direct recognition information between a target and a probe on the sensor surface. Therefore, SPR sensor enables monitoring the real-time interaction among molecules on a tiny chip which can be a powerful analytical tool for purpose of medical diagnostics, food safety analysis and environmental monitoring [19, 93].

A competitive-type SPR sensor for DON and OTA detection by imaging nanoplasmonics was developed [94]. A portable nanostructured imaging surface plasmon resonance (iSPR) chip was immobilized with 3-dimensional carboxymethylated dextran. Afterwards, target mycotoxins and immobilized mycotoxins competitively bound to the corresponding antibodies producing SPR signals. The SPR-based sensor achieved portable detection for DON and OTA in beer with the LOD of 17 and 7 ng/mL, respectively.

Another study described a SPR-based aptasensor in a direct assay format for AFB1 detection. The aptamers were modified on the commercial sensor chip surface, and the SPR signals increased when AFB1 bound to aptamers to achieve quantification with the LOD of 0.4 nmol/L (124.8 pg/mL) [95]. A label-free microfluidic SPR biosensor based on nanoparticles integrated gold chip for Aflatoxin B1 detection was developed. A self-assembled monolayer (SAM) Au chip was modified with AFB1 antibodies and functionalized lipoic acid AuNPs were deposited on it. This multilayer functionalized AuNPs modified Au chip exhibited better sensing performance than bare self-assembled Au chip with the LOD of 0.003 nmol/L (0.93 pg/mL) [96].

By comparing these studies, we can infer that direct SPR-sensors are simpler and more sensitive than indirect SPR-sensors. However, because the small molecule analytes (such as mycotoxins) cannot cause obvious mass concentration changes at the sensor surface, the direct SPR-sensor’s chip surface must be modified with noble metal NPs for signal amplification. More advanced portable SPR instruments are required for practical application.

Optical biosensor platforms that recently reported for mycotoxin determinations are listed in Table 2.

**Table 2:** Summary of optical biosensor platforms for detecting mycotoxins

Target	Principle	Materials	Sample	LOD	Ref.
OTA/AFB1	Colorimetric	Aptamer, Fe_3_O_4_/graphene oxide and Fe_3_O_4_@Au	Agricultural products	0.5/5 ng/mL	[76]
AFB1/AFG1/AFM1/OTA/ZEN	Colorimetric	gold and silver nanoparticles	Pistachio, wheat, coffee and milk	2.7/ 7.3/ 2.1/3.3/ 7.0 ng/mL	[77]
AFB1	Colorimetric	Aptamer and AuNPs	Animal feed and milk	10 nmol/L	[97]
ZEN	Colorimetric	Aptamer and AuNPs with peroxidase-like activity	Corn and corn oil	10 ng/mL	[98]
AFB1/OTA	Fluorescent	Aptamer, DNA tweezers	Food	0.035 ng/mL	[83]
AFB1/OTA/FB1	Fluorescent protein microarray	Antibody, TiO_2_-modified porous silicon	Rice, corn and wheat	0.243/0.433/0.093 ng/mL	[99]
ZEN/OTA/FB1	Fluorescent	Aptamer, upconversion nanoparticle and gold nanoparticle	Corn	30/10/0.02 pg/mL	[100]
ZEN/FB1	Fluorescent	Aptamer, gold nanorods and upconversion nanoparticles	Corn	0.01/0.003 ng/mL	[101]
AFB1/FB1	Fluorescent	Aptamer, graphene oxide/Fe3O4 and CdTe quantum dots	Peanut	6.7/16.2 pg/mL	[85]
OTA/OTB	Fluorescent	Nanobody, Eu/Tb nanosphere	Rice	0.06/0.12 ng/mL	[20]
FB1/OTA	Fluorescent	Aptamer, time-resolved nanoparticles and magnetic nanoparticles	Maize	0.019/0.015 pg/mL	[81]
OTA/AFB1	Fluorescent	Aptamer, SiO_2_@QDs and magnetic nanoparticles	Corn	0.067/1.7 pg/mL	[102]
OTA/ZEN	Fluorescent	Antibody, upconversion-encoded microspheres and phycoerythrin	Corn	0.34/0.41 ng/mL	[82]
AFB1/ZEN	Fluorescent quenching	Aptamer, AuNCs and WS_2_ nanosheet	Maize	0.34/0.53 pg/mL	[84]
ZEN/T-2/AFB1	Fluorescent quenching	Aptamer, time-resolved nanoparticles and WS_2_ nanosheet	Maize	0.51/0.33/0.40 pg/mL	[103]
AFB1	Fluorescent quenching	Aptamer, CdZnTe QDs and AuNPs	Peanut	20 pg/mL	[79]
OTA	Fluorescent quenching	Aptamer, nitrogen doped carbon dots and AgNPs	Flour and beer	8.7 nmol/L	[80]
AFB1/OTA	Chemiluminescence	Antibody and AgNPs	Red yeast rice	0.44/0.83 pg/mL	[87]
DON	Electrochemiluminescence	Antibody, NPCo/Co_3_O_4_–Au and RuSi@Ru (bpy)_3_^2+^	Wheat flour	1 pg/mL	[104]
OTA	Electrochemiluminescence	MIP, CdTe QDs and [Ru (bpy)_3_]^2+^	Starch	0.25 fg/mL	[90]
AFB1	Electrochemiluminescence	Antibody and ZnCdS@ZnS quantum dots	Lotus seed	0.01 ng/mL	[92]
AFM1	Electrochemiluminescence	Aptamer, AuNPs-magnetic nanoparticles and luminol-functionalized silver nanoparticle-decorated graphene oxide	Milk	0.05 ng/mL	[105]
OTA	Electrochemiluminescence	CdS QDs, Cy5-labeled DNA	Beer and wine	0.012 nmol/L	[91]
AFB1/OTA/ZEN/DON	SPR	Antibody and self-assembled monolayer SPR chips	Cereal	0.59 /1.27 /7.07/ 3.26 ng/mL	[106]
DON/OTA	SPR	Antibody, nanostructured gold chips	Beer	17/7 ng/mL	[94]
AFB1	SPR	Antibody, AuNPs and self-assembled monolayer Au chips	Wheat	0.003 nmol/L	[96]
AFB1	SPR	Antibody, gold chips	Grains	2.51 ppb	[107]
AFB1	SPR	Aptamer, gold chips	Red wine	0.4 nmol/L	[108]

#### Electrochemical biosensors

Electrochemical biosensors are based on changes in outputted electrical signals that produced by the chemical reactions between electrode-immobilized recognition elements and target analytes. According to the types of detectable electrical signals, electrochemical sensors can be categorized into amperometric, potentiometric, conductometric, impedimetric and voltammetric methods. Besides, the electrode system (working electrode, reference electrode and counter electrode) is vital to the sensors because identification of target analytes needs to be finished on the electrode. The electrode is also used to conduct electrical signals [12, 109].

Traditional electrochemical immunosensors using general electrodes have some defects of low sensitivity, low stability, and inability to detect multiple targets simultaneously. To address these issues, improving performance of working electrodes and achieving signal amplification have become necessary. Commonly used working electrodes such as glass carbon, gold, and silver electrodes have recently been modified with various nanomaterials (carbon nanotubes, graphene oxide, metal nanoparticles, thin-layer MoS_2_, and porous metal organic framework) to increase their surface area [110–113]. As a result, the nanostructured rough electrode surface can be immobilized with more recognition reagents and more sufficient contact with the analytes so that sensitivity and conductivity are greatly improved [114]. For example, a facile electrochemical immunosensor was constructed for rapid detection of ZEN using thin-layer molybdenum disulfide and thionin composites (MoS_2_-Thi). Thin-layer MoS_2_ is an important graphene analog that is used frequently as a supporting substrate for stabilizing nanoparticles [112]. In this research, ZEN monoclonal antibodies were modified with platinum (Pt) nanoparticles then immobilized on MoS_2_-Thi composites to obtain synergistic signal amplification. Therefore, this immunosensor was easy to operate as well as offered a higher sensitivity compared with the original ZEN monoclonal antibodies.

Screen-printed electrodes have become popular for their advantages of reliability, reproducibility, ductility, ease of mass production, and low costs. They are variable in shape and small enough to be combined with miniaturized devices. Similarly, screen-printed electrodes are also modified with various nanomaterials to enhance sensitivity of electrochemical sensors [115–117].

Microfluidic systems employed in electrochemical immunosensor is an ideal choice to reduce detection time, improve stability and detect multiple targets simultaneously [118]. For example, Lu et al. [119] reported a dual-channel microfluidic electrochemical immunosensor for FB1 and DON detection in corn. Three-electrodes were etched on transparent indium tinoxide (ITO)-coated glass. A sample solution was introduced in the capillary-driven polydimethylsiloxane (PDMS) microfluidic channel. This microfluidic electrochemical immunosensor exhibited high potential for practical application with the LOD of 97 and 35 pg/mL for FB1 and DON, respectively. However, there are few reports on electrochemical immunosensors for detection of multiple mycotoxins.

**Fig. 3:**
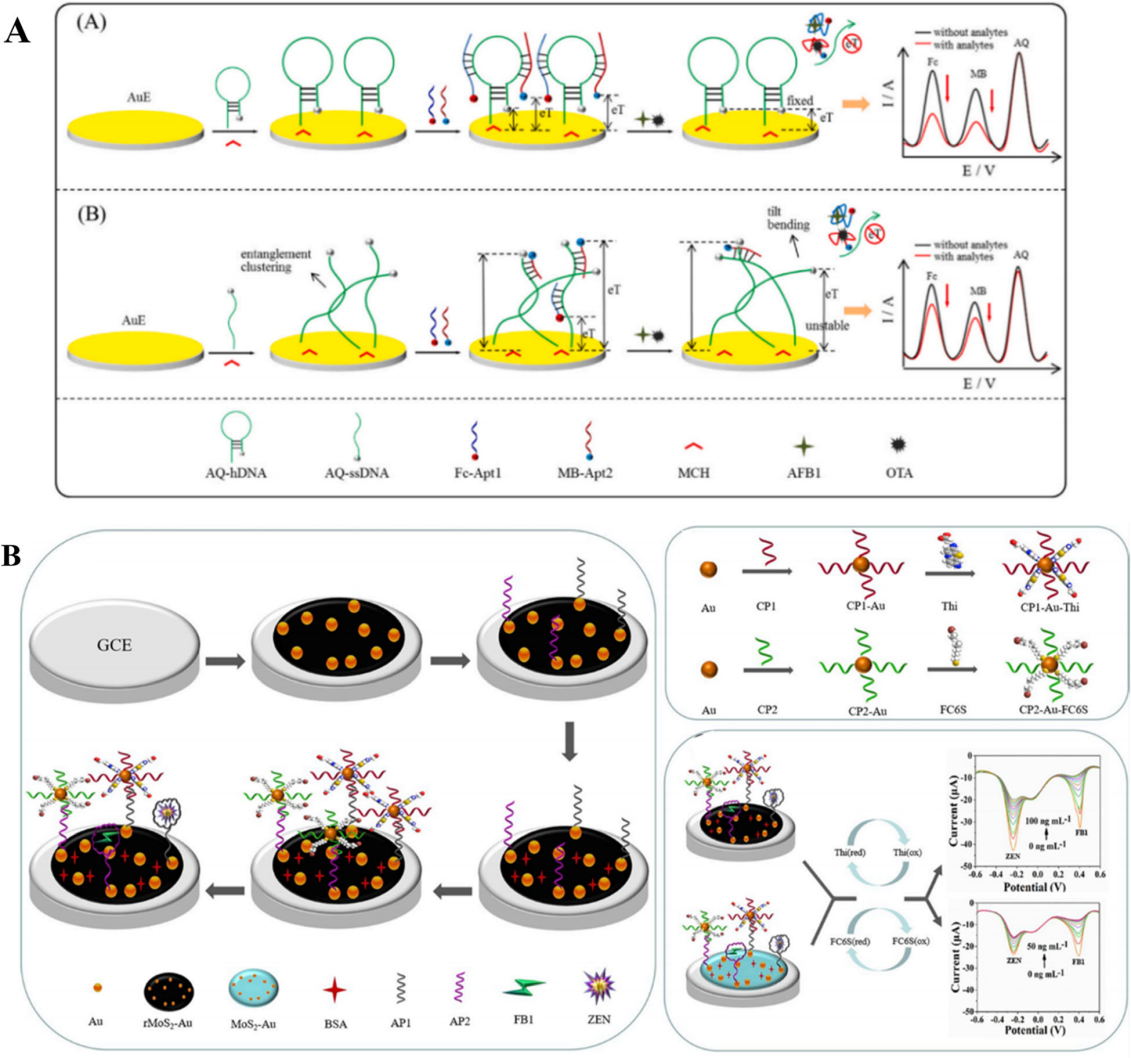
Schematic illustration of the electrochemical sensing platforms. (A) Hairpin DNA-based ratiometric electrochemical aptasensor for simultaneous detection of AFB1 and OTA; (B) dual-target electrochemical aptasensor was developed based on co-reduced molybdenum disulfide and gold nanoparticles (rMoS2-Au) modified electrodes. Diagrams A and B are adapted from Ref. [120] and Ref. [122], respectively

Since antibody’s degradation is still a hinder for regeneration of electrochemical immunosensor sensing elements, aptamer-based sensors (aptasenor) have attracted more and more attention recently. Aptasensors exhibit greater diversity and universality than immunosensors. They are easier to construct for multiple analytes detection systems and sensitivity of aptasensor can be greatly improved by a ratiometric mode. Fig. 3A shows a hairpin DNA-based ratiometric electrochemical aptasensor for simultaneous detection of AFB1 and OTA [120]. Ferrocene-labelled AFB1 aptamer (Fc-Apt1) and methylene blue-labelled OTA aptamer (MB-Apt2) served as binding probes and current signal indicators. Both aptamers were complementary to the carboxylic acid (AQ)-labelled hairpin DNA that served as reference signal. It was applied to analyze AFB1 and OTA in corn and wheat samples with high sensitivity and reliability. Similarly, Wei et al. [121] designed a unique Y-shaped complementary DNA structure for simultaneously hybridizing with OTA and FB1 aptamer. OTA and FB1 aptamers were immobilized with thionine and thiolated ferrocene as signal indicators. It was applied to detect OTA and FB1 in beer with the LOD of 0.47 and 0.26 pg/mL, respectively.

Another dual-target electrochemical aptasensor was developed based on co-reduced molybdenum disulfide and gold nanoparticles (rMoS2-Au) modified electrodes [122]. Aptamers of AFB1 and ZEN were binding with thionine and 6-(Ferrocenyl) hexanethiol modified complementary strands, respectively (Fig. 3B). This platform obtained satisfactory recoveries of AFB1 and ZEN in maize with the LOD as low as 0.4 pg/mL. Li et al. reported a ratiometric electrochemical aptasensor for AFB1 detection in peanut. Ferrocene-labelled aptamer (Fc-apt) served as the response signal and reduced graphene oxide (THI-rGO) functionalized with thionine was used as the reference signal. In this study, ratiometric detection was achieved by formation of Fc-apt-AFB1 complex which resulted in decreased current intensity of Fc (I_Fc_), and increased current intensity of THI (I_THI_) [123]. It is worth mentioning that sensitivity of this sensor can be adjusted by changing the assembly of Fc-apt, which exhibited high sensitivity and selectivity.

Molecularly imprinted polymers (MIPs) can serve as the recognition elements in electrochemical sensors which are favorable alternatives to antibodies. MIPs are mechanical and chemical stable and provide thickness-controlled MIP films on electrode surfaces, which permit direct communication between the films and the transducer. A novel MIP-sensor with two functionalization methods for AFB2 detection in milk was developed. Researches compared ZnO-NPs/chitosan (CS)/AFB2 and ZnO-NPs/Cs/polypyrrole (PPy)/AFB2 composite that electrodeposited on the screen-printed electrode [124]. They showed that ZnO-NPs/CS/PPy/AFB2 functionalized composite had greater sensitivity than ZnO-NPs/CS/AFB2. This MIP-sensor exhibited good specificity, reproducibility and stability as well as extremely low LOD (0.2 fg/mL). Differential pulse voltammetry (DPV) techniques used in this study had a faster and more sensitive response compared to the electrochemical impedance spectroscopy.

In general, electrochemical biosensors show reliable sensitivity and selectivity as well as great potential for miniaturization and portability. But the modification steps involved in the electrochemical biosensors’ preparation are complicated. Reproducibility and interference resistance of traditional electrodes in complex matrices still is problematic. Compared with optical detection methods, there are no huge advantages for development of multiple target detection systems. Table 3 summarizes the important parameters of electrochemical biosensor platforms that have been published in recent years for mycotoxin determinations.

**Table 3:** Summary of electrochemical sensor platforms for detecting mycotoxins

Target	Method	Principle	Electrode	Sample	LOD	Ref.
FB1/DON	Immunosensor	Differential pulse voltammetry	Indium tin oxide electrode integrated with PDMS microfluidic channel	Corn	97/35 pg/mL	[119]
OTA	Immunosensor	Differential pulse voltammetry	Gold electrode	Medicinal and edible malt	0.08 ng/mL	[125]
ZEN	Immunosensor	Square wave voltammetry	MoS_2_-Thi composite-modified glass carbon electrode	Human biofluids	0.005 ng/mL	[112]
ZEN	Immunosensor	Differential pulse voltammetry	Screen-printed electrode	Beer and wine	0.25 ng/mL	[117]
AFB1	Immunosensor	Impedimetric	Cysteine/carbon nanotubes- modified gold electrode	Corn flour	0.79 pg/g	[126]
ZEN	Immunosensor	Differential pulse voltammetry	Multi-walled carbon nanotubes and chitosan-modified glass carbon electrode	Cereal and feedstuff	4.7 pg/mL	[127]
OTA	Immunosensor	Differential pulse voltammetry	Palladium nanoparticles-modified carbon felt electrode	Coffee	0.096 ng/mL	[128]
AFB1	Immunosensor	Cyclic voltammetry	Graphene quantum dots and AuNPs-modified Indium tin oxide electrode	Maize	0.1 ng/mL	[129]
ZEN/FB1	Aptasensor	Differential pulse voltammetry	Co-reduced molybdenum disulfide/AuNPs-modified glass carbon electrode	Maize	0.5 pg/mL	[122]
OTA/FB1	Aptasensor	Differential pulse voltammetry	Gold electrode	Beer	0.47/0.26 pg/mL	[121]
AFB1/OTA	Ratiometric aptasensor	Differential pulse voltammetry	Gold electrode	Corn and wheat	4.3/13.3 pg/mL	[120]
OTA/FB1	Magneto-controlled aptasensor	Square wave voltammetry	Glassy carbon electrode	Maize	20/5 pg/mL	[130]
AFB1	Ratiometric aptasensor	Alternating current voltammetry	Thionine functionalized reduced graphene oxide/AuNPs-modified glass carbon electrode	Peanut	0.016 ng/mL	[123]
T-2	Aptasensor	Differential pulse voltammetry	Molybdenum disulfide/polyaniline/chitosan AuNPs-modified glassy carbon electrode	Beer	1.79 fg/mL	[113]
AFB1	Aptasensor	Square wave voltammetry	Gold electrode	Beer	2 nmol/L	[131]
ZEN	Aptasensor	Square wave voltammetry	Cysteamine-hydrochloride/1,4-phenylene diisocyanate-modified gold electrode	Maize grain	0.017 ng/mL	[132]
AFM1	Aptasensor	Impedance voltammetry	PtNPs/Fe-based metal organic frameworks-modified glassy carbon electrode	Powder and pasteurized milk	2 pg/mL	[133]
ZEN	Molecularly imprinted sensor	Impedimetric	Poly (o-phenylenediamine)- modified screen-printed gold electrode	Corn flakes	0.2 ng/mL	[134]
DON	Molecularly imprinted sensor	Impedimetric	Poly o-phenylenediamine-modified screen-printed gold electrode	Food	0.3 ng/mL	[135]
AFB2	Molecularly imprinted sensor	Differential pulse voltammetry	ZnO-NPs/chitosan/polypyrrole modified screen-printed electrode	Fresh and pasteurized milk	0.2 and 0.7 fg/mL	[124]

### Others

#### Photoelectrochemical sensors

Photoelectrochemical (PEC) sensors are emerging as an analytical technology that combines features of both optics and electrochemistry. In PEC sensors, light source, electrochemical workstation, and signal acquisition system are main components. Light is used as the excitation source and the generated photocurrent is used as the detection signal, which significantly reduces background interference [136]. Photoactive material, as a transducer for the conversion from biological recognition events to observable PEC signals, plays a crucial role in the PEC biosensing platform. Semiconductors (such as QDs) and semiconductor-based heterojunctions (such as graphene and reduced graphene oxide) are commonly used [137–139]. However, PEC biosensors have no obvious advantages over common electrochemical biosensors in sensitivity, simplicity, and practicability at present. Multiple mycotoxins detected by PEC sensors have not been found yet. The detection instruments used for PEC sensor still need a simplification [140].

#### Metal−organic frameworks (MOFs)

In recent years, a novel material has been be applied in fluorescent and electrochemical sensing, termed metal−organic frameworks (MOFs). Metal−organic frameworks are porous coordination polymers with huge surface area, that exhibit excellent optical, electrochemical and catalytic properties [111, 141]. Luminescent metal−organic frameworks (LMOFs), a subclass of MOFs, display outstanding optical properties such as large stokes shifts, high quantum yield, characteristically narrow emission spectra, and long fluorescence lifetime. They also function in recognition and enrichment of targets due to the porous and easily modified structure, which are considered as promising chemical sensor materials [142, 143]. Hu et al. [144] firstly reported a highly luminescent Zn-based metal−organic framework for AFB1 sensing in 2015. The blue luminescence of MOFs can be quenched by AFB1 efficiently and specifically with the LOD of 46 ppb. Another water-stable LMOFs was developed for sensitive and rapid detection of AFB1 in walnut and almond beverages [145]. The LMOF was synthesized by 1,2,4,5-tetrakis (4-carboxyphenyl) benzene (H4TCPB) and highly water-stable Zr, which exhibited raging fluorescence quenching when AFB1 existed with the LOD of 19.97 ppb. These proposed MOFs provide a sensor platform that is very easy to synthesize and operate, but the sensitivity of MOF sensors was not as good as that of other electrochemical and optical biosensors. Anti-interference capability and specific selectivity in complex matrix related to MOFs still requires investigation.

## Conclusion and perspectives

Various mycotoxin contamination occurs frequently and unavoidably in feedstuffs and cereals, which poses enormous risks to public health and leads to economic losses. This paper introduces advanced analytical methods and nanomaterials for mycotoxin detection in recent years including immunoassays and biosensors, especially for multiple mycotoxin detections. Target recognition, signal transduction, and nanoparticles are keys to rapid detection techniques. Analytical methods introduced in this paper are divided into many categories based on these three aspects.

Colorimetric immunoassays are simple, visible, and low-cost methods without the requirement of expensive instruments but the instability, background interference, and poor sensitivity are considerable drawbacks. Therefore, many fluorescent nanoparticles have been introduced to rapid detection and greatly enhance sensitivity and stability. However, some fluorescent signal labels are susceptible to fluorescent bleaching, autofluorescence or environmental interference and require complex labeling steps. Electrochemical biosensors are classic and powerful sensing strategy because of their high sensitivity and ability to be miniaturized, but they are hard to resist interferences, complex electrode modifications are required and electrode fouling and degradation still need to be solved. Photoelectrochemical and electrochemiluminescence sensors are regarded as combining the merits of optical and electrochemical strategies, which show low background, fast response, and high sensitivity. However, the absence of advanced PEC and ECL instrumentation limits practical application. And signal stability and ability to analyze multiple targets of PEC and ECL sensors is not sufficient. Label-free SPR biosensors simplify preparation and detection procedures. They have excellent analytical performance and offers direct and real-time detection platforms by using tiny chips, which make them have great potential for commercial detection of mycotoxins. But SPR sensors are not sensitive to binding events of low molecular weight molecules so that some modifications on chips and sophisticated instruments are still required. Both advantages and disadvantages exist in each kind of detection strategy. We need to choose the appropriate approach according to the conditions and requirements.

In spite of numerous successes with laboratory-based mycotoxin detection, there are still many limitations and challenges to conquer in achieving practical applications.

Firstly, nanomaterials are widely applied almost in all sensing strategies but the function of nanomaterials currently available are not perfect. There are many studies improving material properties and biosensor performances by using hybrid nanostructured materials, but they are not convenient for practical application and mass manufacturing. Developing promising nanomaterials that have excellent optical or electrochemical properties, but also are inexpensive, easy to prepare and environmentally friendly is still a focus of future research. This is also one of the core driving forces behind development of biosensor and immunoassay-based analytical methods.

Secondly, most of the existing sensing strategies are based on single signal output, which are susceptible to instrument conditions and environmental interferences resulting in poor reproducibility and stability. To overcome this drawback, ratiometric sensor with dual signals is an ideal solution. Detection results are based on the ratio of two signals enable self-built-in corrections thus greatly improving sensitivity and accuracy of sensing method. Two different luminescent particles connected together to form a FRET system in optical sensors or two signal labels served as response/reference system in electrochemical sensors are mainstream of ratiometric sensor design. But there are few reports on the detection of mycotoxins by ratiometric biosensors at present.

Lastly, simplicity, sensitivity, and high throughput have not really been implemented simultaneously in a rapid detection method. High-sensitive methods such as electrochemical biosensors often require complex modification operations, while simple and portable LFIA tend to produce qualitative or semi-quantitative detection results. Nowadays, with the development of micro-electro-mechanical systems (MEMS), sensor devices can be miniaturized at a tiny size. There are more and more reports on smartphone-based microfluidic biosensor systems which provide the portable, rapid, and sensitive sensing platforms. This is also a future trend that makes biosensors to better serve humans. Moreover, faced with a wide variety of mycotoxins, there have been many reports on the LFIA strip with multiplex test lines, multiplex SPR biochips, biosensors with multiple labels and integrating biosensors with microarrays and microfluidic systems. Most examples discussed in this paper are appropriate for up to three to six mycotoxins and involved complex designs. But achieving high-throughput quantitative detection of a dozen to dozens of analytes under the premise of guaranteed sensitivity still needs hard work.

Despite many challenges, rapid detection methods based on cross-discipline play an increasingly important role in food safety, industrial manufacture, environmental monitoring, and clinical diagnoses. Traditional determination technology has not been adapted to the demands of rapid, on-site, and large-scale screening. Fast, simple, sensitive, low-cost, and high-throughput analytical methods with portable instrument are growing need. With development of nanomaterials and biosensor technology, rapid detection will receive much more attention in the future.

## Data Availability

Not applicable.

## Abbreviations

AFB1: Aflatoxin B1
OTA: Ochratoxin A
MRLs: Maximum residue levels
ELISA: Enzyme-linked immunoassay
HPLC: High-performance liquid chromatography
LC-MS/MS: Liquid chromatography-tandem mass spectrometry
MS: Mass spectrometry
MIPs: Molecularly imprinted polymers
SELEX: Systematic evolution of ligands exponential enrichment
SPE: Solid phase extractions
NIPs: Non-imprinted polymers
FLISA: Fluorescent-linked immunosorbent assays
CLEIA: Chemiluminescence enzyme immunoassays
LFIA: Lateral-flow immunoassays
POCT: Point-of-care testing
AuNPs: Gold nanoparticles
FB1: Fumonisin B1
ZEN: Zearalenone
FMs: Fluorescent microspheres
CNPs: Carbon-based nanoparticles
TRFMs: Time-resolved fluorescent microspheres
CG: Colloidal gold
PMs: Polystyrene microspheres
SERS: Surface-enhanced raman spectroscopy
IFE: Inner-filter effect
FRET: Förster resonance energy transfer
DON: Deoxynivalenol
T−2: T-2 toxin
SPR: Surface plasmon resonance
MNPs: Magnetic nanoparticles
GO: Graphene oxide
QDs: Quantum dots
UCNPs: Upconverting nanoparticles
AuNCs: Gold nanoclusters
LOD: Limit of detection
PEC: Photoelectrochemical
MOFs: Metal−organic frameworks
